# Mid-term Body Mass Index increase among obese and non-obese individuals in middle life and deprivation status: A cohort study

**DOI:** 10.1186/1471-2458-5-32

**Published:** 2005-04-05

**Authors:** Georgios Lyratzopoulos, Patrick McElduff, Richard F Heller, Margaret Hanily, Philip S Lewis

**Affiliations:** 1 Directorate of Clinical Services and Public Health, Norfolk, Suffolk and Cambridgeshire Strategic Health Authority, Fulbourn, UK; 2 Evidence for Population Health Unit, University of Manchester, Manchester, UK; 3 Former Stockport Healthcare NHS Trust, Stockport, UK; 4 Stockport NHS Trust, Stockport, UK

## Abstract

**Background:**

In the UK, obesity is associated with a clear socioeconomic gradient, with individuals of lower socioeconomic status being more likely to be obese. Several previous studies, using individual measures of soecioeconomic status, have shown a more rapid increase in Body Mass Index (BMI) over time among adults of lower socioeconomic status. We conducted a study to further examine whether ecologically defined deprivation status influences within-individual BMI change during middle life, as the answer to this question can help determine optimal preventive strategies both for obesity *per se*, and its' associated socioeconomic disparities.

**Methods:**

Anonymised records of participants to the Stockport population-based cardiovascular disease risk factor screening programme were analysed. Individuals aged 35–55 who had a first screening episode between 1989 and 1993, and a subsequent screening episode were included in the study. Deprivation status was defined using quintiles of the Townsend score. Mean annual BMI change by deprivation group was calculated using linear regression. Subsequently, deprivation group was included in the model as an ordinal variable, to test for trend. The modelling was repeated separately for individuals who were obese (BMI < 30) and non-obese at the time of first screening. In supplementary analysis, regression models were also adjusted for baseline BMI.

**Results:**

Of 21,976 women and 19,158 men initially screened, final analysis included just over half of all individuals [11,158 (50.8%) women and 9,831 (51.3%) men], due to the combined effect of loss to follow-up and incomplete BMI ascertainment. In both sexes BMI increased by 0.19 kg/m^2 ^annually (95% Confidence Intervals 0.15–0.24 for women and 0.16–0.23 for men). All deprivation groups had similar mean annual change, and there was no evidence of a significant deprivation trend (p = 0.801, women and 0.892, men). Restricting the analysis to individuals who were non-obese at baseline did not alter the results in relation to the lack of a deprivation effect. When restricting the analysis to individuals who were obese at baseline however, the findings were suggestive of an association of BMI increase with higher deprivation group, which was further supported by a significant association when adjusting for baseline BMI.

**Conclusion:**

In the study setting, the BMI of non-obese individuals aged 35–55 was increasing over time independently of deprivation status; among obese individuals a positive association with higher deprivation was found. The findings support that socioeconomic differences in mean BMI and obesity status are principally attained prior to 35 years of age. Efforts to tackle inequalities in mean BMI and obesity status should principally concentrate in earlier life periods, although there may still be scope for focusing inequality reduction efforts on obese individuals even in middle life.

## Background

Increased attention is being paid to the "epidemic of obesity" in post-industrialised countries [1-3]. Since the 1980s, there has been a rapid rise in the proportion of the population who are obese, and in the mean values of Body Mass Index (BMI) and other anthropometric measurements associated with obesity. One of the striking characteristics of the epidemic is that it appears to affect most age groups, including children [4-8].

Among individuals of middle age, obesity is one of the cardiovascular risk factors that demonstrate a clear socioeconomic gradient, with individuals of lower socioeconomic status being more likely to be obese [9,10]. At least four previous studies have shown that individuals with lower socioeconomic status experience a more rapid BMI increase during adulthood [11-14]. These previous studies measured socioeconomic status directly, using either job grade [11], own or parental occupation-defined social class [12,13], employment status [12], or educational attainment [12-14]. These studies also included individuals whose minimum baseline age was 25 years or younger [11-14]. It is therefore important to further examine whether cross-sectional socioeconomic gradients in BMI progress continuously throughout the life course, including during middle life in particular, or whether periods in earlier life are mainly responsible for BMI socioeconomic disparities in later life. As cardiovascular risk greatly increases from middle to late life, determining optimal strategies for the prevention of obesity and its' associated socioeconomic disparities in middle-aged individuals is of great importance to public health and healthcare policy-making.

We therefore examined mean annual BMI change among participants of a primary care based cardiovascular risk factor screening programme co-ordinated by a UK Health Authority. The Stockport Cardiovascular Disease Risk Factor Screening Programme was originally introduced in 1989. Using a call-recall system operated by the Stockport Health Authority, residents registered with a Stockport General Practitioner (GP) and aged between 35 and 60 were invited five-yearly to book a screening appointment. During the 1989–1993 period, about 10.8% of all patients that were registered with a GP were excluded from initial screening invitation, as they were already known to suffer from hypertension (3.91%), diabetes (1.23%) and conditions including history of any cardiovascular disease, and terminal illness (6.64%) (see Additional File 1).

In relation to all Stockport residents aged 35–60 enumerated at the 1991 Census the population coverage was 53.7% for women and 47.5% for men, however true coverage among invitees was higher as the denominator used in this calculation also includes those individuals excluded from screening invitation (see also Additional File 1). Coverage was significantly higher among least deprived groups in men (but not in women), however the magnitude of this difference was small (i.e. coverage of 49.4%, 48.3%, 48.4%, 46.5% and 44.8% for the least to the most deprived quintile group respectively, p < 0.001 (see Additional File 1 for details of the method used for this calculation). Because exclusions due to already known (treated) hypertension or cardiovascular disease can be hypothesized to be more frequent among more deprived individuals (due to a higher prevalence of hypertension and cardiovascular disease in those individuals), the true coverage among deprived invitees may be higher to that calculated, as the denominator used includes "excluded" cases. Individual data on risk factor levels were collated by the Health Authority and anonymised into a usable electronic dataset, used in the present study. An evaluation of the quality and utility of this dataset for research purposes was carried out in 2002. The evaluation concentrated on examining data completeness, and whether or not there were systematic differences in completeness of data items by individual patient characteristics. Also, whether the sample of screened individuals was concordant to the socio-demographic characteristics of the Stockport population of similar age. The evaluation concluded that the quality of the data was excellent [15].

## Methods

Individuals who attended for an initial screening during the first five-year cycle of the screening programme (1989–1993) and who also attended for screening on a subsequent occasion to the end of 1999 were included in the study. Analysis was restricted to individuals who at the time of the first screening were aged 55 years or younger, as older individuals would have not usually been scheduled for a subsequent 5-yearly screening episode (the upper age limit for participation to the Programme being 60).

### Measurements

On both screening occasions height was measured to the nearest centimetre and weight to the nearest kilogram. Weight scales and stadiometers of variable types were used, available at the Stockport General Practice surgeries participating in the scheme. Protocols were in place and training was provided about measurement of weight and height by a visiting nurse screening facilitator, whose role was to quality assure and co-ordinate the implementation of the screening activity. Weight was measured with light clothing and without shoes. Body Mass Index was calculated by dividing weight in kilograms by height in meters squared. Other measurements are described in Additional File 1.

Information on deprivation status was based on the Townsend deprivation score of the enumeration district of residence (1991 Census) [16]. The Townsend deprivation score measures deprivation at a small area level (Census Enumeration District), using information from four separate Census-based variables: unemployement (unemployed residents aged over 16 as a percentage of all economically active residents aged over 16), overcrowding (households with one person per room and over as a percentage of all houselhods), non-car ownership (households with no car as a percentage of all households), and non-home ownership (households not owning their own home as percentage of all households) (s ). Five deprivation quintiles were defined, using the participant's distribution of the Townsend score. The range of Townsend scores among study participants was -7.12 to +10.9 (mean: -1.69, median: -2.39, standard deviation: 2.88, quintile-defining points: -4.10, -2.93, -1.77, +0.61). The range for the population of England and Wales as a whole was -7.55 to +11.8 (mean: 0, median: -0.65, standard deviation: 3.39, quintile-defining points: -3.1, -1.55, 0.39 and 3.13).

### Statistical analysis

Whether "loss-to-follow-up" (i.e. lack of second screening) was differential was examined using multiple logistic regression. The dependent binary variable was attendance (or not) for a second screening; and the independent variables were age, presence of risk factor values above cut-off points and deprivation group. Similarly, whether completeness of BMI ascertainment (i.e. whether BMI was measured on both the first and the second screening) was differential was examined using multiple logistic regression.

The significance of baseline deprivation group differences in obesity status (BMI >30) and mean BMI value was assessed with simple logistic and linear regression respectively, entering deprivation group as a continuous variable.

To examine mean annual BMI change during follow-up, a linear regression model was fitted with change in BMI between the initial and second screening as the dependent variable and follow-up time in years (taken as screening interval between the two screening episodes) as the independent variable of interest, adjusting for age at initial screening (model 1). In this model, the co-efficient for follow-up time denotes the mean (age-adjusted) annual BMI change per year of follow-up. The model was subsequently fitted to each deprivation group stratum separately (model 2), producing five different co-efficients for follow-up time, denoting the mean (age-adjusted) annual BMI change for each deprivation group. To test for a statistically significant effect of deprivation on BMI change, all individuals were subsequently included in one model and deprivation group entered as a continuous variable (and age- and follow-up period-adjusted) (model 3). In this model, the co-efficient for deprivation group denotes the difference in BMI change for each one level increase in deprivation group.

### Stratified analysis by baseline obesity status

The analysis was repeated stratifying by baseline obesity status, i.e. sequentially restricting the analysis to individuals who were obese and non-obese at baseline (BMI values of 30+ or <30 respectively).

### Baseline BMI adjustment

Furthermore, as per previous research on the subject, [11-14] analysis was repeated adjusting for baseline BMI (i.e. at the first screening) in all models.

## Results

In total there were 21,976 women and 19,158 men aged 35–55 who had a first screening episode during 1989 and 1993, of whom 16,932 women (77%) and 13,812 men (72.1%) also had a second screening. Deprivation status was available for 99.8% of all cases. The mean screening interval (follow-up period) was 4.79 years for women and 4.83 years for men. Persons lost to follow-up were significantly more likely to be obese, hypertensive, to have high cholesterol, and to be current smokers, younger and more deprived (Table 1).

**Table 1:** Baseline characteristics and completeness of follow-up (second screening). Significance levels from multiple regression models.

**Baseline characteristic**	**Women (n = 21,976)**			**Men (n = 19,158)**		
	
	**Initial screen only**	**Two screens**	**p**	**Initial screen only**	**Two screens**	**p**
**Mean age (years)**	44.5	44.9	<0.001	44.3	44.7	<0.001
**% current smokers**	39.9	36.4	0.018	53.4	48.0	<0.001
**% with SBP>140 mmHg**	25.5	18.2	<0.001	33.2	24.8	<0.001
**% with DBP>90 mmHg**	15.3	9.4	*	23.9	17.0	*
**% cholesterol > 6.5 mmol/l**	25.5	22.6	0.019	36.7	29.8	<0.000
**% BMI >30 kg/m**^2^	17.7	11.7	<0.000	15.9	11.9	0.001
**% in the two most deprived groups (4 and 5)**	43.6	37.3	<0.001**	42.7	36.8	<0.001**

SBP: Systolic Blood Pressure; DBP: Diastolic Blood Pressure

*Significant in multiple logistic regression model excluding SBP but omitted from presented model to avoid over-adjustment, as highly correlated with SBP.

** Deprivation group (quintile) entered as a continuous variable.

Among individuals with two screening episodes 11,158 women (65.9%) and 9,831 men (71.2%) had dual (i.e. in both screens) BMI ascertainment. Individuals were significantly more likely to have complete BMI ascertainment if they were more deprived; and for men only, if they were current smokers and of younger age (Table 2). Hypertensive men and women, and obese men were significantly less likely to have complete ascertainment. High cholesterol at baseline did not have a significant effect.

**Table 2:** Baseline characteristics and completeness of "dual" BMI ascertainment (i.e. BMI measurement on both screening episodes). Significance levels from multiple regression models.

	**Women (n = 16,932)**			**Men (n = 13,812)**		
	
	**Without "dual" BMI ascertainment**	**Dual BMI ascertainment**	**p**	**Without "dual" BMI ascertainment**	**Dual BMI ascertainment**	**p**
**Mean age (years)**	45.3	44.6	0.717	45.3	44.2	0.001
**% current smokers**	14.3	36.1	0.980	18.6	44.4	0.024
**% with SBP>140 mmHg**	23.0	15.7	<0.001	30.9	22.3	<0.001
**% with DBP>90 mmHg**	12.0	8.1	*	21.7	15.1	*
**% cholesterol > 6.5 mmol/l**	7.6	15.2	0.064	11.5	21.2	0.253
**% BMI >30 kg/m^2^**	4.8	11.4	0.150	6.0	11.2	<0.001
**% in the two most deprived groups (4 and 5)**	29.6	41.2	<0.001**	31.7	38.8	0.006**

SBP: Systolic Blood Pressure; DBP: Diastolic Blood Pressure

*Significant in multiple logistic regression model excluding SBP but omitted from presented model to avoid over-adjustment, as highly correlated with SBP.

** Deprivation group (quintile) entered as a continuous variable.

From the original cohort, final analysis included just over half of all individuals (50.8% of women and 51.3% of men), due to the combined effect of loss to follow-up and incomplete BMI ascertainment. The combined effect of differential loss to follow-up and BMI ascertainment completeness in relation to deprivation group is summarised in Additional File 2.

Among individuals with dual BMI ascertainment, at the initial screening, 11.4% of women and 11.2% of men were obese (BMI > 30 kg/m^2^). The percentage of women who were obese was 7.8%, 8.6%, 9.3%, 13.1% and 17.6% for the least to most deprived quintiles, respectively (p < 0.001). For men the respective percentages were 8.8%, 9.3%, 11.2%, 11.4% and 15.7% (p < 0.001). The overall mean BMI was 24.79 kg/m^2 ^for women and 25.69 kg/m^2 ^for men. The mean levels of BMI for women (initial screening) were 24.15 kg/m^2^, 24.35 kg/m^2^, 24.67 kg/m^2^, 25.05 kg/m^2 ^and 25.74 kg/m^2 ^for the least to most deprived quintiles, respectively (p < 0.001); and for men 25.41 kg/m^2^, 25.56 kg/m^2^, 25.79 kg/m^2^, 25.70 kg/m^2 ^and 26.07 kg/m^2 ^respectively (p < 0.001).

**Figure 1:**
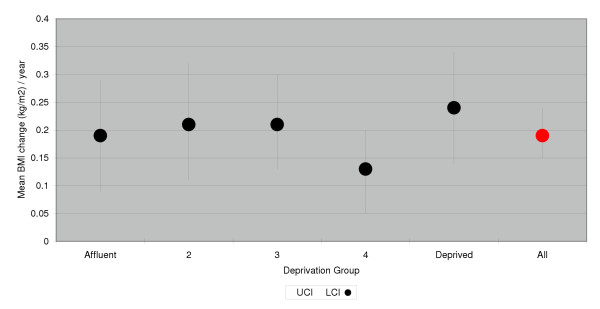
Mean annual change in BMI (age- and follow-up- adjusted), women.

The average annual increase in BMI among women was 0.19 kg/m^2 ^(95% Confidence Intervals [CI]: 0.15–0.24), and 0.19 kg/m^2^, 0.21 kg/m^2^, 0.21 kg/m^2^, 0.13 kg/m^2 ^and 0.24 kg/m^2 ^for least to most deprived quintiles respectively (Table 3 and Figure 1). In men, the average annual increase was also 0.19 kg/m^2 ^(CI: 0.16–0.23) and 0.22 kg/m^2^, 0.16 kg/m^2^, 0.15 kg/m^2^, 0.24 kg/m^2 ^and 0.18 kg/m^2 ^for least to most deprived quintiles respectively. There were no significant trends in mean annual BMI change across deprivation groups (p = 0.801, women, 0.891, men).

**Table 3:** Mean annual Body Mass Index change, adjusted for age and follow-up time.

**All individuals**									
		**Women**				**Men**			
		
		**Mean**	**LCI**	**UCI**	**p**	**Mean**	**LCI**	**UCI**	**p**

**All (Women: n = 11,158, Men: n = 9,831)**	***Model 1***								
	**All**	0.19	0.15	0.24	<0.001	0.19	0.16	0.23	<0.001
	
	***Model 2***								
	**Affluent**	0.19	0.09	0.29	<0.001	0.22	0.15	0.30	<0.001
	**2**	0.21	0.11	0.32	<0.001	0.16	0.09	0.23	<0.001
	**3**	0.21	0.13	0.30	<0.001	0.15	0.08	0.22	<0.001
	**4**	0.13	0.05	0.20	0.001	0.24	0.18	0.31	<0.001
	**Deprived**	0.24	0.14	0.34	<0.001	0.18	0.11	0.26	<0.001
	
	***Model 3***								
	**Trend**	0.00	-0.04	0.03	0.801	0.00	-0.03	0.03	0.891

**Stratified analysis to non-obese individuals**									

**BMI < 30 at first screening (Women: n = 9,891, Men: n = 8,729)**	***Model 1***								
	**All**	0.19	0.15	0.23	<0.001	0.16	0.13	0.19	<0.001
	
	***Model 2***								
	**Affluent**	0.19	0.10	0.27	<0.001	0.18	0.12	0.24	<0.001
	**2**	0.24	0.15	0.32	<0.001	0.12	0.05	0.18	<0.001
	**3**	0.20	0.12	0.28	<0.001	0.13	0.07	0.19	<0.001
	**4**	0.13	0.05	0.20	0.001	0.21	0.15	0.27	<0.001
	**Deprived**	0.22	0.13	0.31	<0.001	0.13	0.06	0.21	<0.001
	
	***Model 3***								
	**Trend**	0.00	-0.03	0.03	0.954	-0.01	-0.04	0.01	0.352

**Stratified analysis to obese individuals**									

**BMI 30+ at first screening (Women: n = 1,267, Men: n = 1,102)**	**Model 1**								
	**All**	0.16	-0.03	0.36	0.107	0.34	0.19	0.49	<0.001
	
	**Model 2**								
	**Affluent**	0.24	-0.59	1.07	0.570	0.39	-0.08	0.87	0.106
	**2**	0.04	-0.62	0.71	0.902	0.30	-0.06	0.66	0.105
	**3**	0.29	-0.18	0.75	0.229	0.25	-0.12	0.63	0.185
	**4**	0.09	-0.18	0.37	0.505	0.39	0.10	0.69	0.008
	**Deprived**	0.28	-0.07	0.63	0.120	0.38	0.11	0.65	0.005
	
	**Model 3**								
	**Trend**	0.18	-0.01	0.37	0.057	0.17	0.00	0.33	0.049

Model 1. Adjusted for age and follow-up time, with all participants included. Reported co-efficient of Model 1 denotes mean annual BMI change (kg / m^2 ^/ year).

Model 2. As for model 1, but stratified by deprivation group. Reported co-efficients denote the mean annual BMI change for each deprivation group.

Model 3. As for model 1, but deprivation group entered as ordinal variable, with all individuals included. Reported co-efficient denotes the additional mean annual BMI change for each one level increase in deprivation group (e.g. from deprivation group i to i+1).

LCI: Lower Confidence Interval; UCI: Upper Confidence Interval, BMI: Body Mass Index.

### Analysis stratified by baseline obesity status

Restricting the analysis to individuals who were not obese at the time of first screening produced trivial changes to the results by deprivation group, and once more there was no significant deprivation trend in mean annual BMI change (p = 0.954, women, p = 0.352, men) (Table 3). The small sample size limits the precision of estimates obtained when restricting analysis to individuals who were obese at baseline, however a borderline significant effect of deprivation on BMI change was found in test for trend for both men and women (p = 0.057 for women, and 0.049 for men).

### Adjustment for Baseline BMI

When adjusting for baseline BMI value (i.e. at screening episode 1) results in models 1 (mean annual change for all individuals) and 2 (mean annual change by deprivation group) change little (Table 4). However, for women only, the test for trend (model 3) shows a significantly higher mean annual BMI increase with higher deprivation quintile.

**Table 4:** Mean annual Body Mass Index change, adjusted for age, follow-up time and baseline BMI value (screening episode 1).

**All individuals**									
		**Women**				**Men**			
		
		**Mean**	**LCI**	**UCI**	**p**	**Mean**	**LCI**	**UCI**	**p**

**All (Women: n = 11,158, Men: n = 9,831)**	***Model 1***								
	**All**	0.18	0.14	0.22	<0.001	0.18	0.14	0.21	<0.001
	
	***Model 2***								
	**Affluent**	0.18	0.08	0.28	<0.001	0.19	0.12	0.26	<0.001
	**2**	0.21	0.11	0.31	<0.001	0.14	0.07	0.21	<0.001
	**3**	0.21	0.12	0.29	<0.001	0.13	0.06	0.20	<0.001
	**4**	0.12	0.04	0.19	0.003	0.23	0.17	0.29	<0.001
	**Deprived**	0.21	0.11	0.30	<0.001	0.18	0.10	0.25	<0.001
	
	***Model 3***								
	**Trend**	0.04	0.01	0.07	0.016	0.01	-0.01	0.04	0.331

**Stratified analysis to non-obese individuals**									
**BMI < 30 at first screening (Women: n = 9,891, Men: n = 8,729)**	***Model 1***								
	**All**	0.19	0.15	0.23	<0.001	0.16	0.13	0.19	<0.001
	
	***Model 2***								
	**Affluent**	0.18	0.10	0.27	<0.001	0.17	0.11	0.24	<0.001
	**2**	0.24	0.15	0.32	<0.001	0.12	0.05	0.18	<0.001
	**3**	0.20	0.12	0.27	<0.001	0.12	0.07	0.18	<0.001
	**4**	0.13	0.05	0.20	0.001	0.21	0.15	0.27	<0.001
	**Deprived**	0.21	0.12	0.29	<0.001	0.13	0.06	0.20	<0.001
	
	***Model 3***								
	**Trend**	0.01	-0.02	0.04	0.619	-0.01	-0.04	0.01	0.331

**Stratified analysis to obese individuals**									

**BMI 30+ at first screening (Women: n = 1,267, **Men: n = 1,102)	**Model 1**								
	**All**	0.16 0.03	-	0.36	0.107	0.34	0.19	0.49	<0.001
	
	**Model 2**								
	**Affluent**	0.24	-0.50	0.98	0.522	0.48	0.13	0.83	0.008
	**2**	0.29	-0.25	0.82	0.293	0.21	-0.12	0.54	0.208
	**3**	0.32 0.14	-	0.78	0.172	0.15	-0.16	0.47	0.336
	**4**	0.08	-0.20	0.36	0.570	0.34	0.06	0.61	0.016
	**Deprived**	0.20	-0.12	0.53	0.222	0.46	0.23	0.70	<0.001
	
	**Model 3**								
	**Trend**	0.20	0.03	0.37	0.024	0.21	0.07	0.36	0.003

Models 1–3 notes: As explained at footnote of Table 3, but all models adjusted for baseline BMI

LCI: Lower Confidence Interval; UCI: Upper Confidence Interval, BMI: Body Mass Index

When stratifying the (baseline-BMI-adjusted) analysis to individuals who were non-obese at baseline a significant effect of deprivation status on mean annual BMI change could not be shown (Table 4). Inversely, a significant effect of deprivation on BMI change was found among individuals who were obese at baseline (p = 0.024 for women and 0.003 for men).

## Discussion

This study shows increasing BMI trends among adult UK individuals of both sexes who were followed up for a mean of 4.8 years chiefly during the late 1980s to mid-1990s. Although in cross-sectional analysis participants manifest the well-known socioeconomic pattern of obesity, a deprivation status effect on mean annual BMI change could not be shown, except for individuals who were obese at baseline. This finding is suggestive that the socioeconomic gradients in mean BMI and obesity in middle aged individuals are principally determined by factors operating prior to age 35, with the exception of obese individuals, in whom deprivation group differentials on BMI increase appear to continue even during middle age.

The results of this study in part contrast with previous research demonstrating that lower socioeconomic status is associated with accelerated weight gain during adulthood [11-13], or at least during part of adulthood [14]. When interpreting such differences, methodological differences in a wider range of factors, including the study population, the age of participants, the length of follow-up, the measurement (and number of measurements) of BMI, the measurement of socioeconomic status, and analytical approaches used should be taken into consideration (Table 5). In our opinion the key difference between the present and the above studies relates to the minimum baseline age of participants, which in this study is considerably higher (35 years) compared to all previous studies. The findings of the present study do not suggest that socioeconomic differentials in BMI change do not exist in general, rather that among non-obese individuals these may cease to exist after the age of 35.

**Table 5:** Comparative features of previous and current study about effect of socioeconomic status on BMI change.

**Ref.**	**Population (% coverage)**	**No of individuals included in final analysis (% women)**	**Min-Max age at baseline**	**Min-Max follow-up (mean follow- up)**	**No of BMI measurements/method**	**SES measurement**	**Whether baseline BMI adjustment (or otherwise taken into account) in analysis**	**Main findings in relation to SES effect on BMI change**	**Comment**
11	Subset of "Whitehall II" civil servant cohort study (73%, actual coverage higher as ~4% of invited persons had moved)	2,466 W 5,507 M (30.9%)	25 y – 25 y	~25 y – ~25 y (~25 y)	2 / 1^st ^recalled, 2^nd ^measured	Employment grade (I-III)	Yes	Significant SES effect, particularly among those with largest BMI increase (i.e. > 6 kg/m^2^)	Individuals who lost weight / BMI during follow-up were excluded.
12	Subset of Malmo Diet and Cancer Study, excluding those with history of cancer, heart attack and stroke inter alia. Initial "invited" sample random. (NR)	5,464 W (100%)	20 y – 20 y	25 y – 53 y (36.6 y)	2 / 1^st ^recalled, 2^nd ^measured	Employment status Own occupational group Paternal (bread-winner) occupational group Educational attainment	Yes	Significant SES effect, for all different SES measures	
13	Subset of the Medical Research Council National Survey of Health and Development Cohort Study (socially stratified cohort of 1946 newborns)	2,659 M + W (% W not explicitly reported in this study, originally cohort 47.5%)	20 y – 20 y	6 y (f-up 1)-23 y (f-up 4) (NR)	4 / First 2 recalled (some indication of underestimate), last 2 measured	Paternal Social Class at age 14 Also Educational attainment	Yes	Significant childhood SES effect, even adjusting for educational attainment	
14	Finnish Twin Cohort Study (89% to 1^st ^questionnaire, follow-up q'rres coverage of 84% and 77%)	2,482 monozygotic and 5,113 dizygotic twin pairs (56% of participants were W)	18 y – 60 y	6 y (f-up 1) 15 y (f-up 2) (6 y and 15 y for f-up 1–2 respectively	3 / All self-reported (validation study proves good validity)	Educational attainment	Yes	Significant SES effect for BMI change between 1975–1981, but no effect between 1981 and 1990	
PS	Borough residents (53.7% W 47.5% M, actual coverage higher as 10.8% "excluded" cases also included in denominator)	11,158 W 9,831 M (53.1% W)	35 y – 55 y	1 y – 10 y (4.8 y)	2 / Both measured	Ecological (based on small area deprivation measurements)	Yes	Null SES effect for non-obese individuals, significant SES effect for obese individuals models adjusting for baseline BMI	Stratification of analysis by baseline obesity status

Ref: Reference, No: Number, BMI: Body Mass Index, SES: Socioeconomic Status, PS: Present study, W: Women, M: Men, F-up: Follow-up, NR: Not reported, y: years.

A crucial question in relation to the generalisability of the study findings is whether screening participants were representative of the Stockport population as a whole. It has to be borne in mind that persons invited for screening excluded an important proportion of individuals with known hypertension, diabetes and other cardiovascular conditions, i.e. a "healthier" cohort of patients compared to the general population. Overall population coverage was approximately 50%, and reassuringly there were either non-significant (women) or significant but small (men) differences of coverage by deprivation group. Although the retrospective and indirect nature of the assessment of coverage (see Additional File 1) has to be borne in mind, we nevertheless believe that our sample is unlikely to have been substantially different to the overall eligible Stockport population of 35 and 55 year olds. Conservatively, it can as a minimum be assumed that the study is representative of longitudinal BMI changes in middle-aged individuals who at the time of invitation for the first screening were not known not to have an established diagnosis of hypertension, diabetes or cardiovascular disease.

An important proportion of individuals who attended for first screening were "lost to follow-up" and such individuals were more likely to be hypertensive or to have high cholesterol. This most likely reflects the fact that under the operating protocols of the Programme, such individuals were meant to be excluded from further screening invitations, in order to be offered "usual" clinical care for the management of their high cardiovascular risk. The fact that a relatively greater proportion of "lost to follow-up" individuals were obese and deprived could have potentially biased the study results. Nevertheless restricting the analysis to participants who were not obese at the initial screening did not alter the principal findings in relation to lack of a deprivation effect on BMI change. Conservatively, in stratified analysis, it could be assumed that the study is representative of longitudinal BMI changes in non-obese (at baseline) individuals.

Dual BMI ascertainment among study participants with two screening episodes was incomplete, and also differential. Individuals that could be perceived to be at higher cardiovascular risk exhibited both higher and lower levels of complete (dual) ascertainment. For example, more deprived individuals and men who were current smokers were more likely to have dual BMI ascertainment, but the opposite was also true for hypertensive individuals, and obese men. Given the non-uniform way by which cardiovascular risk status appears to have influenced dual BMI ascertainment it could be hypothesised that overall this did not introduce an important degree of bias.

Socioeconomic status can be measured directly (i.e. by measuring individuals' income, occupation or education) or indirectly, using area-based measures (i.e. based on the predominant characteristics of the population of a small area) [17]. An area-based measurement (Townsend deprivation score) was used in this study, in common with other previous UK research in the field of socioeconomic inequalities in health, because information about individual measures of socioeconomic status in participants was incomplete, and might have been less accurate. Although in theory the use of a direct individual marker of socioeconomic status may have been preferable, area-based UK deprivation indices have been shown to predict poor health outcomes at the individual level [18], including coronary heart disease [19]. The fact that the area level used was small (Census Enumeration District) diminishes the potential for misclassification error, and a recent study has shown that Enumeration District-based Townsend scores can be a valid measure of individual deprivation [20]. Although Stockport as a whole is less deprived than England and Wales, the range of Townsend deprivation score among study participants was large and comparable to the national range. It is worth noting that at baseline the study participants demonstrate a clear socioeconomic pattern of obesity. This observation supports the validity of deprivation status as an indicator of socioeconomic status among the study's participants.

Was the observed BMI increase among the study participants "natural", or could the dynamics of BMI change of the cohort have been altered by the screening process per se – and if so to what degree? If the latter is true, it is theoretically possible that the observed deprivation group differential in BMI increase among obese individuals were due to those least deprived being differentially more able to more change their dietary or energy expending behaviour following the baseline screening episode attendance. It is impossible to answer the above questions with certainty directly from the information available, and given the uncontrolled nature of the study. We however believe that the findings are much more likely to describe the "natural" BMI increase experience of the cohort, rather than a healthcare-mediated effect for three reasons. Firstly, it is apparent from the findings that screening appears not to have been overall effective in halting a further increase in BMI among obese individuals (Tables 3,4), and this would minimise the theoretical potential for a differential effect of screening by deprivation group. Secondly, although it is not possible to know with certainty how individuals found to be obese at baseline were managed (e.g. advice to lose weight), given the time of the study (1989–1993) it is likely that obesity management would have been given lesser priority as a cardiovascular risk factor, compared with hypertension, smoking and high cholesterol. Thirdly, the evidence overall suggests that, at least in the past, primary care based cardiovascular risk factor screening interventions were of limited effectiveness, [21] although this meta-analysis did not include results about a potential effect of advice on physical activity [22].

Prospective use of routinely collected data has been advocated as a method to help support surveillance and monitoring of risk factor trends in the population [23]. Although a previous meta-analysis or primary studies reported between 1977 and 1996 has questioned the overall efficacy of multiple cardiovascular risk factor screening as a means of preventing the development of cardiovascular disease [21], a potentially important secondary use of population-based screening programmes is as public health surveillance tools, to monitor population risk factor trends. This study proves that indeed there is a potential for using such data for population health monitoring and surveillance, as suggested by both the recent "Wanless Report" [24] and the subsequent UK Department of Health White Paper on Public Health [25]. It is of note that the design and conduct of a population-based cohort study of similar magnitude would have been associated with very considerable resource implications.

## Conclusion

During the study period and in the study setting, the BMI of middle-aged individuals of both sexes residing in a UK district was increasing by 0.19 kg/m^2 ^per year. The increase was similar across individuals of all deprivation groups for non-obese individuals, but was significantly higher among more deprived individuals who were also obese at baseline. The findings support that socioeconomic differences in mean BMI and obesity status are principally attained prior to 35 years of age, although among obese individuals those differentials may be further augmented during middle life. Efforts to tackle inequalities in obesity status should therefore principally concentrate in earlier life periods. Increasing BMI trends in individuals of middle age are likely to have important detrimental and multiplicative effects on the overall population burden of cardiovascular risk factors and population health in future years.

## Competing interests

The author(s) declare that they have no competing interests.

## Authors' contributions

GL identified the research question with support from RFH. GL has developed the statistical methodology, with supervisory support by PMcE. PL and MH have over a number of years helped run the Stockport Cardiovascular Risk Factor Screening Programme and collect and collate data that enabled the analysis to be carried out. GL wrote the first draft of the manuscript. All authors contributed to the writing of the paper and read and approved the final manuscript. The work leading to this report has been carried out as part fulfillment for the study for the degree of MD, University of Manchester, for GL. RFH is GL's supervisor for the named degree and P McElduff GL's advisor.

**Figure 2:**
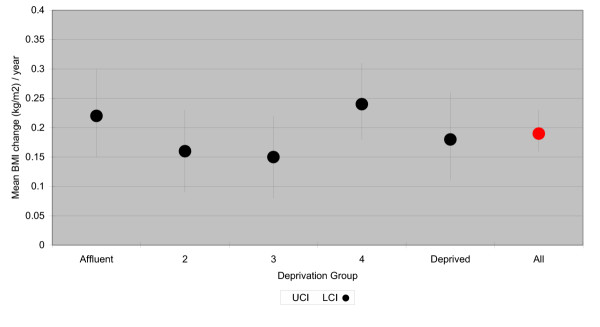
Mean annual change in BMI (age- and follow-up- adjusted), men.

## Pre-publication history

The pre-publication history for this paper can be accessed here:



## Supplementary Material

Additional File 1 **Supplementary information about the Stockport Cardiovascular Risk Factor Screening Programme 1989–1993. **This file provides additional information about operational aspects of the Stockport Cardiovascular Risk Factor Screening Programme, including about exclusions from screening invitations, measurements of risk factors, and overall population coverage. Click here for file

Additional File 2 **Synthesis of information presented in **Tables 1,2, **in relation to deprivation group. **This file provides information about the combined effect of loss to follow up and incomplete "dual" (i.e. on both screening episodes) ascertainment of BMI by deprivation group, synthesising relevant information from the Results section and Tables 1 and 2 Click here for file
