# Supplementary material for: Mid-term Body Mass Index increase among obese and non-obese individuals in middle life and deprivation status: A cohort study
Source: BMC Public Health. 2005 Apr 5;5:32. doi: 10.1186/1471-2458-5-32 (PMC1090593; doi:10.1186/1471-2458-5-32)
Supplement: Additional File 1 — Supplementary information about the Stockport Cardiovascular Risk Factor Screening Programme 1989–1993. This file provides additional information about operational aspects of the Stockport Cardiovascular Risk Factor Screening Programme, including about exclusions from screening invitations, measurements of risk factors, and overall population coverage. [file 1471-2458-5-32-S1.doc]

**Additional File 1. Supplementary information about the Stockport Cardiovascular Risk Factor Screening Programme 1989-1993**

**1. Exclusions**

It is customary for centrally co-ordinated but practice-based screening programmes to provide the opportunity to front-line screening staff [e.g. General Practitionerss (GPs)] to “correct” the invitation list by excluding patients for whom screening would be judged inappropriate or useless. For example, in the UK, women with previous hysterectomy are excluded from invitation for a cervical smear screening. Similarly in the case of the Stockport CVD RFS programme, before screening invitations were sent out to patients of a particular GP, “prior notification” was given to the screenees’ GP, who had the option to within 28 days return to the Health Authority a modified list. In the modified lists, GPs could “exclude” patients from a screening appointment invitation. As alluded in the text, during the 1989-1993 period, about 10.8% of all patients that were registered with a GP were excluded from initial screening invitation, as they were already known to suffer from hypertension (3.91%), diabetes (1.23%) and conditions including history of any cardiovascular disease, and terminal illness (6.64%).

**2. Population coverage (general and by deprivation group)**

There was no prospective information available about the number of persons “screened” as opposed to persons “invited”. Therefore, coverage by sex, for the population as a whole and for each Townsend deprivation index quintile was assessed retrospectively with the following method.

*Numerator information*

Programme participants (first screening) during the “prevalence round” 1989-1993 were used as the numerator. Deprivation group status was ascribed to each participant as described in the main article (quintiles of Census Enumeration District Townsend deprivation index score).

*Denominator information*

The number of Stockport residents aged 35-64 in each ED of the 1991 census was obtained from the “MIMAS Census Dissemination Unit CASWEB” search engine (University of Manchester). Data were stratified by sex 5-year age bands. The number of 60 year-old residents, was assumed to equate the number of residents in the age band 60-64 divided by 5. This was subsequently added to the number of individuals in all other 5-year old band (35-59) to produce the number of 35-60 year olds who were resident in each Stockport ED in the 1991 census –as this was the age group targeted by the Programme.

Using the same Townsend deprivation ED score quintile defining points as for the numerator, the 589 Stockport EDs were split into deprivation quintiles, and for each sex, the number of individuals aged 35-60 in each deprivation quintile was calculated.

*Coverage calculation*

For each sex, the coverage by deprivation quintile was subsequently calculated as the number of screening participants in each quintile divided by the respective number of individuals resident in the EDs of the respective quintile. The chi-squared test for trend was used to assess significance of any deprivation trends in screening coverage, using STATS DIRECT. The results are shown below.

Additional File 1 Table: Population coverage of the screening Programme 1989-1999

|  | Women aged 35-60 | | Men aged 35-60 | |
| --- | --- | --- | --- | --- |
| Deprivation Group | Coverage (%) | Test for trend | Coverage (%) | Test for trend |
| Affluent | 54 | p=0.55 | 49.4 | p<0.001 |
| 2 | 53.7 |  | 48.3 |  |
| 3 | 54.1 |  | 48.4 |  |
| 4 | 53.3 |  | 46.5 |  |
| Deprived | 53.3 |  | 44.8 |  |
| All | 53.7 |  | 47.5 |  |

It is worth noting that true coverage would have been higher, as the denominator used in the above calculation includes all residents, i.e. even those that would have been excluded due to established / known cardiovascular disease or risk factors (see above).

**3. Other measurements**

Throughout the study period, total cholesterol was measured to second decimal point level of accuracy in mmol/l at the biochemistry laboratory of the Stepping Hill Hospital (a typical UK District General Hospital) by an enzymatic colorimetric assay using cholesterol esterase and cholesterol oxidase.[[1]](#endnote-2) Throughout the study period the laboratory participated in external quality assurance schemes. Fresh blood samples were sent to the laboratory and processed within 24 hours from sampling.

Systolic and diastolic blood pressure were measured with mercury sphygmomanometers of variable types available at the Stockport General Practice surgeries participating in the scheme, to a standardised protocol conforming with the 1987 “Recommendations on Blood Pressure Measurement” of the British Hypertension Society.[[2]](#endnote-3) Training about measurement of blood pressure to doctors and practice nurses was provided by a visiting nurse facilitator, employed by the Stockport Health Authority, whose role was to quality assure and co-ordinate the implementation of the screening activity. Systolic blood pressure value was defined as the value at which the I^st^ Korotkoff sound becomes audible, and diastolic blood pressure was defined as the value at which the V^th^ Korotkoff sound becomes inaudible, both recorded to the nearest 2mmHg (i.e. mercury column drop speed of 2mmHg/sec). A minimum of two measurements were made and the reported values represent the mean of the separate measurements.Error: Reference source not found

Smoking status was measured by direct questioning (self-reported). For the purposes of this analysis a dichotomous variable (current vs. non-current smoker) was created, but merging the “previous smoker” and “never smoker” categories.

1. Allain CC, Poon LS, Chan CS, Richmond W, Fu PC. Enzymatic determination of total serum cholesterol. Clin Chem. 1974;20(4):470-5. [↑](#endnote-ref-2)
2. British Hypertension Society. Recommendations on Blood Pressure Measurements. BMJ. 1987. [↑](#endnote-ref-3)
