# Supplementary material for: Mid-term Body Mass Index increase among obese and non-obese individuals in middle life and deprivation status: A cohort study
Source: BMC Public Health. 2005 Apr 5;5:32. doi: 10.1186/1471-2458-5-32 (PMC1090593; doi:10.1186/1471-2458-5-32)
Supplement: Additional File 2 — Synthesis of information presented in Tables 1,2, in relation to deprivation group. This file provides information about the combined effect of loss to follow up and incomplete "dual" (i.e. on both screening episodes) ascertainment of BMI by deprivation group, synthesising relevant information from the Results section and Tables 1 and 2 [file 1471-2458-5-32-S2.doc]

Additional File 2 Figure 1: Effect of deprivation group on loss to follow-up, and loss of information due to the combined effect of loss to follow-up and incomplete “dual” (on both screens) BMI ascertainment. Women (n=21,976)

Additional File 2 Figure 2: Effect of deprivation group on loss to follow-up, and loss of information due to the combined effect of loss to follow-up and incomplete “dual” (on both screens) BMI ascertainment. Men (n=19,158)
